# Hypothermia among trauma patients in the Emergency Department (ED): a review of documentation and management

**DOI:** 10.1186/1757-7241-22-S1-P10

**Published:** 2014-07-07

**Authors:** Lucy V Harten-Ash, Anthony Hudson

**Affiliations:** 1 Emergency Department, St. Georges Healthcare NHS Trust, London, UK

## Background

As a component of the lethal triad in trauma; hypothermia is associated with a significantly worse outcome. It is an independent indicator of mortality, and early recognition and aggressive management is therefore critically important. St. Georges is a major trauma centre and currently sees around 120 trauma patients per month. We designed an audit to determine our current practice of temperature measurement in trauma, and to evaluate our management of hypothermia in trauma.

## Method

A retrospective audit of 93 case notes. Data was collected from all patients triaged to the ED as “Major trauma” during a three month period (October-December 2012 inclusive).

## Results

24% of trauma patients arrived with a pre-hospital temperature recorded. 67% had a primary survey temperature; of these 31% had no further temperature recorded. 17% of patients did not have any recorded temperatures during their time in the ED.

37% had a documented temperature of <36 whilst in the ED.

52% of patients had a warming method documented.

Of the hypothermic patients; normothermia was achieved in 44%. 22% left the ED with a greater temperature than on arrival but remained hypothermic. In 3% there was a documented fall in temperature.

## Discussion

Hypothermia is common among our trauma patients. We feel that it is currently undervalued by our trauma team members. There is substantial room for improvement in our recording of temperatures. We are currently achieving normothermia in less than half of the hypothermic trauma patients.

Since our audit we have provided education on the significance of hypothermia and how to manage it in trauma. We have acquired a heated mattress for use in these patients. We have written a “Temperature in Major Trauma” guideline, and amended the trauma pro-forma to increase compliance with regular measurement, and to improve management of hypothermia.
